# Don’t Worry, We’ll Call You: An Anticipatory Guidance Framework for Addressing Test-Related Anxiety—Scanxiety

**DOI:** 10.2196/89201

**Published:** 2026-11-05

**Authors:** Justin Ross, Liz Salmi, Chen-Tan Lin

**Affiliations:** 1 Integrative Medicine, University of Colorado Health, 8111 E Lowry Blvd #120, Denver, CO, 80230, United States, 1 3033321206; 2 Open Notes Lab, Beth Israel Deaconess Hospital, Boston, MA, United States; 3 Department of Medicine, University of Colorado Health, 8111 E Lowry Blvd #120, Denver, CO, 80230, United States, 1 3033321206

**Keywords:** patient-physician communication, patient portal, open notes, open results, patient engagement, patient anxiety, electronic health record, test result management

## Abstract

The 21st Century Cures Act information blocking rule that went into effect in 2021 in the United States requires that health systems not interfere with delivering test results to patients, including laboratory, pathology, and imaging results. Consequently, many health systems release results immediately, and patients often view results before the ordering clinician has seen them. Patients overwhelmingly want this access. In a 2023 multisite survey, 95.7% (7520/7859) of patients preferred immediate release. Yet the same survey found that 16.5% (403/2442) of patients receiving abnormal results reported increased worry from reading them before clinician contact. In this viewpoint, written with a patient coauthor living with a grade 2 astrocytoma, we argue that there are 2 distinct forms of anxiety. The distress of serious medical news is unavoidable. The anxiety of not knowing what a phrase means or when someone will contact the patient can be reduced. We propose that clinicians address the second form of anxiety at the moment of test ordering rather than when the result arrives. We identify 3 sources of distress when a result appears in a portal: threat, uncertainty, and loss of control. We use a real radiology result, “suspicious for tumor progression,” and the patient coauthor’s experience of the distress that patients call “scanxiety,” to illustrate these themes in an anticipatory guidance conversation. Borrowing the framework of anticipatory guidance from pediatric practice, we offer a 5-step conversation guide for clinicians at the time of test ordering: (1) inform the patient they are likely to see the result before the clinician can contact them, (2) tell the patient they have a choice whether to look immediately, (3) mention there are trusted resources for the patient to read about the result, (4) describe to the patient the best and worst case scenarios and how they would be contacted in each case, and (5) solicit questions. We pair this with recommendations for health systems and electronic health record vendors, principally that portals distinguish “you have a new test result” from “you have a message from your team” and that notification settings be easy to silence. We believe immediate patient access to test results is the right direction. We propose that anticipatory guidance is an important framework to reduce a major component of test-related anxiety and that it should be widely adopted by clinicians.

## Introduction

Cancer diagnoses are bad news. Even more so, the anxiety surrounding a cancer diagnosis is magnified when the patient does not understand the report and it is unclear when a clinician will contact them about next steps. The first situation is unavoidable, while the second is entirely avoidable. In an ideal situation, patients would receive a biopsy, have it immediately reviewed, and then a trusted clinician would deliver the information in-person. However, in the context of the 21st Century Cures Act information blocking rule, immediacy is unrealistic for most clinicians. The rule, effective in April 2021 in the United States, prohibits health care organizations from interfering with patients’ access to their electronic health information [1,2]. In practice, laboratory, pathology, and imaging results are released to the patient portal as soon as they are finalized, and routinely before the ordering clinician has seen them. The old sayings “don’t worry, we’ll call you” and “no news is good news” are no longer acceptable practice. A new approach is needed for communicating with patients about the release of test results. This viewpoint has two aims: (1) to explain why the immediate release of test results creates a form of anxiety that is separate from the news itself; and (2) to offer clinicians a short, specific conversation—delivered when the test is ordered and not when the result arrives—that reduces that anxiety. We propose a 5-step guide for clinicians and companion recommendations for health systems and electronic health record (EHR) vendors.

## Patient-Partnered Case Study

LS is a 42-year-old woman with grade 2 astrocytoma. She is clinically doing well and continues to recover from surgery. Unfortunately, there are concerning changes in her imaging results (Textbox 1).

Textbox 1. **Actual radiology result.**

Impression: since [January], new enhancing nodular FLAIR [fluid-attenuated inversion recovery] hyperintensity at the anterior aspect of the left parietal resection cavity, suspicious for tumor progression.

The patient expresses how she feels in the following comment.

*I’ve lived with a slow-growing, malignant brain tumor (grade 2 astrocytoma) for more than a decade. The issue of recurrence isn’t a matter of if, but rather, when. When it comes to scan-related anxiety—or “scanxiety,” as it’s better known among us patients—it doesn’t matter how informed I am, I still feel it. Despite best efforts, I know that much of the cancer experience is out of my control.*

*For me, the optimal waiting period between scan and answer should be as short as possible. Do I want the results shared with me in a face-to-face discussion with my oncologist? That is preferred. But would I prefer to wait 24 hours or longer for that answer to be delivered personally? Absolutely not.*

[LS]

The term “scanxiety” was coined in 2011 by the writer and cancer survivor Bruce Feiler, describing the distress surrounding an imaging test in post-cancer follow-up, before the scan and until the result is announced [3]. It has since entered into use in peer-reviewed literature, including 2 scoping reviews and an analysis of how patients discuss scanxiety with one another on social media [4-6].

After the 21st Century Cures Act went into effect in 2021, LS and millions of other US adults had immediate access to their test results through online patient portals. In a 2023 multisite survey of patients and care partners, most respondents (7520/7859, 95.7%) preferred to receive results immediately, including 2337 of 2453 (95.3%) who received results they perceived as not normal [7]. Overall, few respondents (411/5473, 7.5%) reported that reviewing results before a clinician contacted them increased their worry. However, that figure was considerably higher among those receiving abnormal results (403/2442, 16.5%) than among those whose results were normal (294/5918, 5.0%; odds ratio 2.71, 99% CI 1.96‐3.74). An earlier 2013 study published by Kaiser Permanente found similar results [8].

There is tension in the drive toward information immediacy and transparency vs the consequences of unintended psychological harm. Scan-associated anxiety is common and has been characterized in peer-reviewed literature, including 2 scoping reviews of adults with cancer [4,5]. Its scale is also visible in portal behavior. In an analysis of 968,774 results reviewed by 290,349 patients, patients repeatedly refreshed the portal while awaiting 250,796 (25.9%) results overall, including 25,280 of 64,356 (39.3%) results from tests that the authors classified as high sensitivity. This was a pattern the investigators interpret as a potential marker of worry during the waiting period [9]. There have also been anecdotal reports of increased anger, anxiety, and stress among patients receiving bad news through immediate test result access [10]. The American Medical Association reports that a number of states are moving to enable exceptions to the federal rule on prohibition of information blocking [11].

How might we help LS and her oncologist resolve this common scenario? We believe there is an effective way forward.

## Anticipatory Guidance Framework

The framework of anticipatory guidance can help. This term has been used in pediatric literature to indicate guidance that a provider gives a parent as their child grows, including common expectations about development in the coming months and years [12]. In the case of LS and others, we use the term to forecast possible patient reactions to an unknown test result as a means of reducing associated anxiety.

Test-related anxiety is not a disease. Instead, scanxiety is a normal reaction to a potentially life-threatening situation. As medical professionals, we have a duty to validate, to understand, and to improve this experience for patients.

Patients who receive test results without context through online patient portals face 3 risks.

1. *Threat state*. Anxiety arises from real or perceived threat. The greater the threat, the more likely the anxiety. Thus, anxiety is magnified when test results pose a threat to a person’s health or life.
2. *Uncertainty*. Patients experience uncertainty in 3 ways. There is uncertainty over the meaning of medical terms, uncertainty over the timing of discussing the results with their provider, and uncertainty of what their treatment choices are.
3. *Lack of control*. Patients experience lack of control when they receive test results at unpredictable times with notifications that may be unwanted and when they cannot reach their doctor immediately to discuss the results.

When considering patient LS, her situation contains all 3 risks. “Suspicious for tumor progression” is a threat to health and to life. Uncertainty appears twice: in the meaning of “new enhancing nodular FLAIR [fluid-attenuated inversion recovery] hyperintensity” and in not knowing when her oncologist will call. Lack of control is in the timing: the result arrived when the radiologist signed it, not when she was ready to read it. Anticipatory guidance offered when the scan was ordered would not have changed the finding; yet, it would have addressed the second and third risks.

Efforts to reduce test-related anxiety have concentrated on how a result is presented once it has been released; a 2024 systematic review found that presentation format measurably affects patient understanding [13]. Less attention has been given to what is said before the test is ordered. Notably, the 2023 survey found no association between precounseling and reduced worry (odds ratio 0.70, 99% CI 0.31‐1.59) [7]. That null result described existing practice: “precounseled” was an unstructured and undefined yes/no survey item covering everything from a thorough conversation to a passing remark. Specifying what the conversation should contain is precisely the gap this framework fills.

## Patient Perspective

The patient shares her perspective on offering guidance:

*As a veteran portal user, I’m fine reviewing results online while waiting for a more formal discussion with my oncologist. However—as a patient advocate who connects across diverse patient communities—I’ve learned not all people wish to receive results at the same pace as myself.*

*Before ordering any new test, clinicians should offer guidance about what a patient can expect when results are released to online portals. A conversation as simple as the following could be deployed at the time the test is ordered: “I’m ordering a test. You might see the result before me. Worst case, this could be X. and this is how I would reach you. Best case, this could be Y. and this is how we would reach you. What questions do you have?*”

[LS]

## Recommendations for Clinicians

Clinicians should adopt anticipatory guidance as a standard process when ordering tests to help patients manage threat, reduce uncertainty, and regain agency (Textbox 2). Clinicians can empathize with patients that receiving test results via the portal can be anxiety-producing, and that this is a normal human reaction. Clinicians can assure patients, “We will work together on this.” Clinicians can also state how and when the patient can expect contact with the health care team to reduce uncertainty. Clinicians can remind patients that they have control over when to view results, including their ability to turn off notifications in the portal and reduce unwanted early notifications. The intensity of anticipatory guidance should scale with the stakes of the test. A routine lipid panel may warrant no more than a brief statement that results will appear in the portal; complex imaging, genetic testing, and pathology warrant the full conversation in Textbox 2.

Textbox 2. **Anticipatory guidance: 5-step conversation guide for clinicians and patients.**

- I am ordering a test. *You may see results before I can contact you.*
- *You have a choice*: look immediately or wait to hear from me.
- *If you choose to look*: medlineplus.gov is a trusted resource [14].
- *Here are the best and worst cases*, and how we would contact you.
- *What questions do you have?*

## Recommendations for Health Systems and EHR Vendors

EHR systems should implement features on patient portals that make it as easy for patients to “turn off notifications” as it is to silence a cell phone. Further, portal notifications should distinguish between “You have a new test result” and “You have a message from your provider/team” [15]. This way, patients can choose to ignore the automated result and wait for a personal message. We believe a better understanding of a person’s control will be beneficial for patients who experience increased worry when viewing test results, without impairing the experience of patients who prefer immediate access to results. We also recommend all clinicians move the discussion on the possible meaning of test results to the time of test ordering. This can be done with a brief anticipatory guidance discussion (Textboxes 2 and 3) at the time testing is conducted, rather than waiting until results are available (Multimedia Appendices 1 and 2).

Textbox 3. **Anticipatory guidance: guidance and proposed policy for health systems.**

- *Inform clinicians and staff*: test results deliver to the patient portal immediately.
- *Pre-visit testing*: make it easy for clinicians to order tests *before* appointments.
- Develop and distribute the *1-page anticipatory guidance education for clinicians*.
- Develop and distribute the *1-page anticipatory guidance education for patients* at laboratory draw stations, radiology, etc.
- *Add anticipatory guidance language* to the results section of the patient portal.
- Make it easy for patients to *adjust their notification settings* within the portal.

Anticipatory guidance is not equally available to every patient. Those with limited English proficiency, limited health or digital literacy, unreliable internet access, cognitive impairment, or high baseline anxiety may be the least equipped to interpret an unaccompanied result and least likely to find a notification setting to change. Some rely on a caregiver to read results. Guidance offered at the time of ordering must therefore be delivered in the patient’s language and at an accessible reading level, and health systems should not treat portal notification controls as a substitute for direct contact with patients who are unlikely to use a portal at all.

Anticipatory guidance will not change the fact that some health news will be serious or life-threatening. However, this framework offers an opportunity to understand the patient experience and reduce unintended negative impact to patients as they receive unfiltered test results and wait for guidance from their care team.

Whether anticipatory guidance changes what patients experience is an empirical question. The outcomes most worth measuring are patient-reported anxiety while awaiting results, the interval from result release to clinician contact, and the volume of patient-initiated messages after release.

We want to be clear about what this framework does and does not do. It addresses the avoidable anxiety of uncertainty and lack of control: not knowing what a word means or what happens next. It will not reduce the distress of serious medical news.

## Conclusions

The immediate release of test results to patients improves information transparency and timeliness. At the same time, it could increase the anxiety of patients receiving unfiltered results via the patient portal. Anticipatory guidance for test results is a 5-step conversation guide that can reduce patient anxiety about next steps (Textbox 2). A patient who reads “suspicious for tumor progression” should feel something. Our aim is that she not also be left alone with it.

## Supplementary material

10.2196/89201 Multimedia Appendix 1 Example of patient education on anticipatory guidance.

10.2196/89201 Multimedia Appendix 2 Example of clinician education on anticipatory guidance.

## Abbreviations

EHR: electronic health record
FLAIR: fluid-attenuated inversion recovery
