# Supplementary material for: Don’t Worry, We’ll Call You: An Anticipatory Guidance Framework for Addressing Test-Related Anxiety—Scanxiety
Source: J Particip Med. 2026 Nov 5;18:e89201. doi: 10.2196/89201 (PMC13739274; doi:10.2196/89201)
Supplement: Supplementary file 1 [file jopm-v18-e89201-s001.pdf]

When Can I See Results in ***My Health Connection***?

News for Patients Getting a Test at UCHealth

YOU can choose when to see your results:

At UCHealth, we believe in building trust and better relationships by sharing your test results with you without delay.

- ***Do you prefer to view test results right away?***
  You may be familiar with medical language. You may have friends or family who can help explain results. You may be comfortable searching the internet to answer questions to confusing or potentially serious results. If so, proceed!

- ***Do you prefer to hear from us first before viewing results?***
  You may want to wait to look at your results online until after we contact you. Usually we will contact you within a few days after the result is available to us.

- ***Sometimes, it is better to get a test done just before your provider visit.***
  Then you can discuss results during your visit. Ask your provider if this might work for you.

MORE ABOUT RESULTS: Some results are ready on the same day; other results may take a few days. **When the result is available, we release them to you online immediately**. Sometimes, this means that you may see your results BEFORE your provider has seen them. Please understand that we look at every test result.

Sometimes, test results may show an exclamation mark (!) or "abnormal" flag. Often, these "abnormal" results do not affect your health. We will always send you a note about what the results mean for you. It may take us 1-2 business days after WE receive the result to send the note to you. For more serious results, we will contact you more quickly.

If you have any concerns, you can **send an online message** by My Health Connection, or by **call our clinic phone number**. You can also turn on/off notifications in the UCHealth app or website by going to **Settings: Notifications**.

Thank you. We are honored that you trust us to be your healthcare partner.
We look forward to working together to keep you as healthy as possible.

2024, UCHealth MHC Team
