# Supplementary material for: Don’t Worry, We’ll Call You: An Anticipatory Guidance Framework for Addressing Test-Related Anxiety—Scanxiety
Source: J Particip Med. 2026 Nov 5;18:e89201. doi: 10.2196/89201 (PMC13739274; doi:10.2196/89201)
Supplement: Supplementary file 2 [file jopm-v18-e89201-s002.pdf]

Sharing Test Results Immediately with Patients: Open Results and the need for Anticipatory Guidance

White paper for Physicians/APPs/Clinical Staff: March 2024, CT Lin & Physician Informatics Group

Physician/APP Informatics Group

We improve physician and team wellness and effectiveness by building extraordinary relationships and innovative tools.

Executive Summary: The Information Blocking Federal Rule (2021) requires release of test results to patients immediately upon request. Most patients want this. For some, this has caused significant anxiety. **Anticipatory Guidance** at the time of placing orders should be the UCHealth standard. Anticipatory Guidance both satisfies the law AND is the right thing for patients. Here is why and how.

Background: The 21st Century CURES Act makes it illegal for health systems to withhold results from patients by delaying release. UCHealth complied with the law in 2021.

- **CT scan showing cancer**. Dr. Robert Breeze, neurosurgeon, uses anticipatory guidance. At time of ordering the test (CT, MRI, etc), he explains that the patient may see the result before he will. He explains the range of possible results and the actions they will take. Consequently, the timing of release and the next conversation is not time-critical, and patients appreciate the forethought. It is not more work, it is time-shifted upstream.
- **Data**. A four-health-system study* in 2022 (including UCHealth) of 8000 patients receiving test results online indicated that **96% of patients seeing results online (normal or abnormal) WANTED to continue to receive results immediately**. 8% indicated they worried more.
- **Threat, Uncertainty and Lack of Control** are what patients face when seeing new diagnoses and results. From the literature, the concept of **Anticipatory Guidance** addresses these concerns up front and reduces uncertainty.

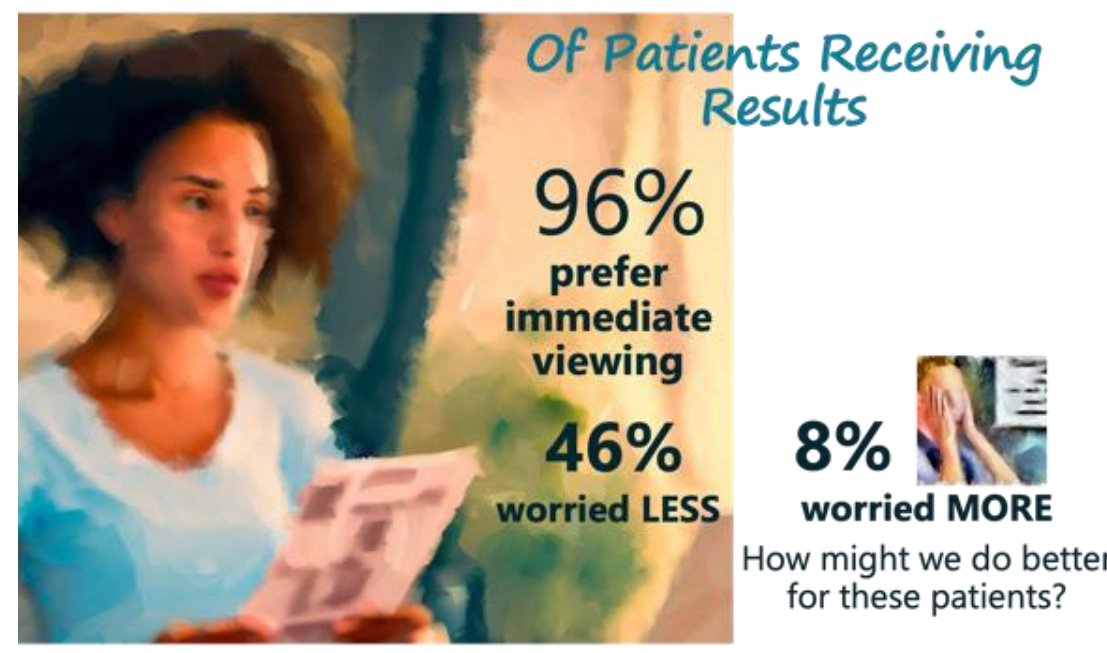

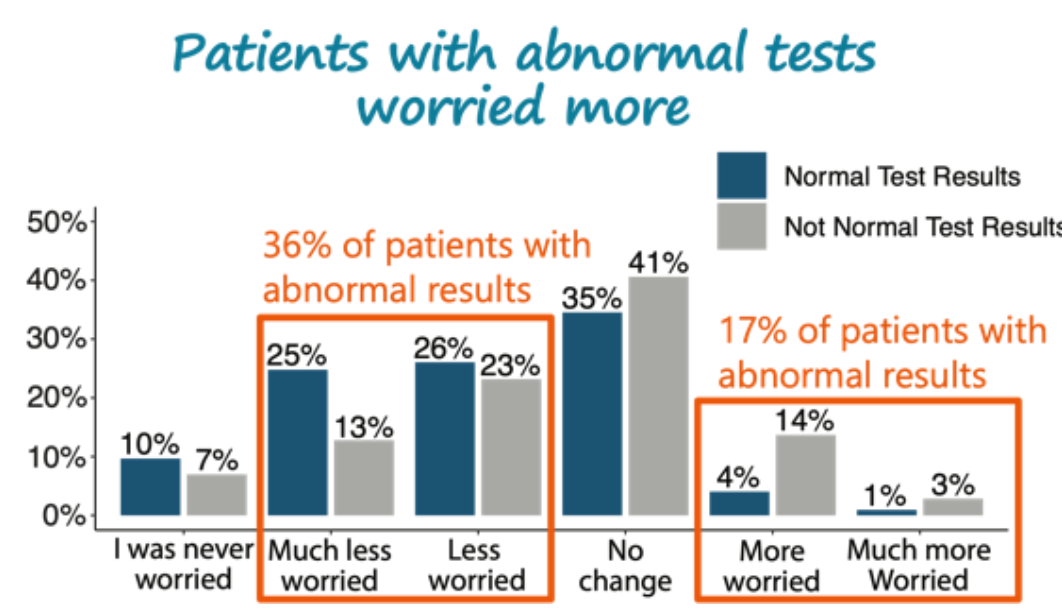

Assessment/Recommendations

For Staff (RNs, MAs): "We release all test results online to you. But, everyone is different. You can choose to look up the result right away or wait for us to contact you. ***Medlineplus.gov*** is an excellent online tool about test results." Also, **you can turn on/off your notifications** in your UCHealth app or UCHealth My Health Connection site (Settings: Notifications, Communications)

For Providers (MD, DO, APP). We recommend universally using **Anticipatory Guidance** at the time of placing orders as follows:

- ***I'm ordering a test****. You might see the result before me.*
- ***You have a choice****: Look immediately or wait to hear from me.*
- *Here is a trusted resource about test results:* ***medlineplus.gov****.*
- ***Here are the best and worst cases****, and how we would contact you.*
- ***What questions do you have****?*

PROPOSAL

Move the discussion upstream to the time of ordering

PRINCIPLES	ANTICIPATORY GUIDANCE
Reduce Threat and Anxiety	Anticipate range of results
Reduce Uncertainty	Anticipate notification process
Improve Sense of Control	Reinforce Choice. Ask for Q

*Steitz, Turer, Lin, et al. 2023, JAMA Network Open 2023
